# An Assessment of the Serum Activity of ADH and ALDH in Patients with Primary Biliary Cholangitis

**DOI:** 10.1007/s00005-022-00667-4

**Published:** 2022-12-28

**Authors:** Blanka Wolszczak-Biedrzycka, Anna Bieńkowska, Elżbieta Zasimowicz, Grzegorz Biedrzycki, Justyna Dorf, Wojciech Jelski

**Affiliations:** 1 Department of Psychology and Sociology of Health and Public Health, University of Warmia and Mazury, Warszawska 30, 10-082 Olsztyn, Poland; 2 Medical Diagnostic Laboratory, Pro-Medica Hospital, Elk, Poland; 3 Hospital Dispensary, Regional Specialist Hospital, Olsztyn, Poland; 4 Department of Clinical Laboratory Diagnostics, Medical University of Białystok, Białystok, Poland; 5 Department of Biochemical Diagnostics, Medical University of Bialystok, Białystok, Poland

**Keywords:** Primary biliary cholangitis, Alcohol dehydrogenase, Aldehyde dehydrogenase

## Abstract

Primary biliary cholangitis (PBC; previously known as primary biliary cirrhosis) is a chronic inflammation-induced cholestatic process in the liver. Antimitochondrial antibodies (AMAs) are observed in around 90% of patients, which suggests that PBC is an autoimmune disease. Alcohol dehydrogenase (ADH), ADH isoenzymes and aldehyde dehydrogenase (ALDH) are localized in the liver, and they are useful markers of liver dysfunction. In this study, the activity of total ADH, ADH isoenzymes and ALDH was evaluated in the blood serum of patients with PBC. The experimental group comprised 50 PBC patients, both male and female, aged 28–67. The control group consisted of 50 healthy subjects, both male and female, aged 25–65. The serum activity of class I ADH, class II ADH and ALDH was measured by spectrofluorophotometry, whereas total ADH and class III ADH activity was determined by photometry methods. The activity of class I ADH and total ADH was significantly higher in the experimental group than in the control group (*p* < 0.001). An increase in class I ADH and total ADH activity indicates that the isoenzyme class I ADH is released by compromised liver cells and can be useful diagnostic markers of PBC.

## Introduction

Primary biliary cholangitis (PBC) is a chronic autoimmune disease of the liver that leads to the gradual destruction of intra-hepatic bile ducts. The disease is diagnosed based on the serum concentrations of antimitochondrial antibodies (AMAs). Serum AMA titers greater than 1:40 are highly specific indicators of the disease, and they are noted in 90–95% of patients with PBC (Chen et al 2013). Other blood parameters in PBC patients are also indicative of cholestasis. Alkaline phosphatase (ALP) activity is elevated for at least six months. The disease also presents with an increase in gamma-glutamyl transferase (GGT) activity and a moderate increase in aminotransferases: aspartate (AST) and alanine (ALT) levels (Lindor et al. 2009) which do not have diagnostic significance, but could point to the overlap syndrome of autoimmune hepatitis (AIH) with PBC. Similarly to other cholestatic disorders, total serum cholesterol is elevated in PBC. Elevated levels of immunoglobulin M are also a characteristic marker of PBC. A histopathological assessment of liver biopsies could be also helpful in diagnosing PBC, although this procedure is not required according to the recommendations of the European Association for the Study of the Liver (2009).

Numerous studies have demonstrated changes in the activity of alcohol dehydrogenase (ADH), its isoenzymes and aldehyde dehydrogenase (ALDH) in the blood serum of patients with various liver diseases, including hepatitis C, liver tumors, fatty liver disease, and AIH (Chrostek and Szmitkowski 2000; Jelski et al. 2008, 2017, 2018a, 2018b). These liver diseases showed statistically higher activity of class I ADH and total ADH in the serum of patients than in healthy subjects and the results suggest a potential clinical role for ADH (especially class I ADH) as marker for liver disorders. Some authors note that serum total alcohol dehydrogenase activity is an indicator of intra-hepatic cholestasis (Chrostek and Szmitkowski 1997; Mezey and Cherrick 1968). The changes in activity of particular ADH isoenzymes in the sera of patients with different cancers (especially the digestive system: colon, pancreas, esophagus, stomach), seems to be caused by release of this isoenzymes from cancer cells, and may play a potential role as a markers of this cancer (Jelski and Szmitkowski 2008). Alcohol dehydrogenase may also be useful for diagnostics of non-cancerous diseases (e.g., acute pancreatitis, *Helicobacter pylori* infection) (Jelski et al. 2011, 2014). However, the applicability of total ADH, ADH isoenzymes and ALDH in diagnosing PBC has not been evaluated to date. Currently employed screening requires tools and methods that are both highly sensitive and specific when diagnosing the early stages of PBC (e.g., antibodies). Therefore, the aim of this study was to assess the activity of these enzymes in PBC patients and to determine their usefulness as diagnostic markers of the disease.

## Materials and Methods

### Materials

Serum for laboratory analyses was obtained from the venous blood of 50 patients (30 men aged 28–67 and 20 women aged 28–67) who had been admitted to the Clinic of Infectious Diseases and Hepatology of the Medical University of Bialystok (Table 1). Primary biliary cholangitis was diagnosed based on the results of laboratory analyses and a histopathological assessment of liver biopsies. In all patients, serum AMA titers exceeded 1:40, ALP activity exceeded reference values more than fivefold, and aminotransferase and total bilirubin (BilT) activity was somewhat elevated. The patients were divided into four groups characterized by different stages of disease progression based on the grading system proposed by Ludwig and Scheuer (Kakuda et al. 2013): stage 1—portal hepatitis, bile duct damage caused by granulomas (20 patients); stage 2—spread of pathological changes between portal space and bile ducts to liver parenchyma (interface hepatitis), with proliferation of bile ductules surrounding portal space, inflammatory cell infiltration, hepatocellular necrosis (periportal hepatitis) and proliferation of bile ducts (12 patients); stage 3—disruption of hepatic architecture caused by the loss of bile ductules and fibrosis, with numerous fibrous septa (12 patients); stage 4—cirrhosis with the presence of regenerative nodules (6 patients). The exclusion criteria were chronic liver diseases, including drug- or alcohol-induced hepatitis, fatty liver disease, metabolic disorders, genetic disorders, Wilson’s disease and sclerosing cholangitis. All patients tested negative for hepatitis A, B, C, D and E, and they had not consumed alcohol in the past six months (no history of alcohol abuse).

**Table 1:** Characteristic of study group

	Patients with PBC	Control group
Demographic characteristic		
Female	20	25
Male	30	25
Age	28–67	25–65
BMI	< 24.9	< 24.9
Alcohol intake	No	< 20 g of ethanol per week
Laboratory results		
AMA	> 1:40	< 1:40
ALP U/l	2850 ± 45	40 ± 12
ALT U/l	60 ± 13	21 ± 14
AST U/l	55 ± 17	18 ± 12
GGT U/l	30 ± 6	18 ± 12
BilT mg/dl	1.1 ± 0.5	0.6 ± 0.4

The control group consisted of 50 healthy subjects aged 25–65 (25 men aged 25–65 and 25 women aged 28–62). Blood was collected from control group individuals during routine medical examinations at the Occupational Medicine Clinic of the Medical University Hospital in Bialystok. The results of physical examinations and laboratory analyses were not indicative of liver disease in any of the control group subjects (AMA titers and ALP, AST, ALT and GGT activity were within the norm). All control group individuals consumed alcohol only occasionally (< 20 g per week).

### Methods

#### Determination of Total ADH Activity

Alcohol dehydrogenase catalyzes the reduction reaction of p-nitroso-N-N-dimethylaniline (NDMA) by nicotinamide adenine dinucleotide (NADH) that is formed during the enzymatic oxidation of n-butanol in the presence of nicotinamide adenine dinucleotide (NAD^+^). The amount of enzymatically reduced NDMA is a measure of enzyme activity (Orywal et al. 2017).

#### Determination of Class I and Class II ADH Isoenzyme Activity

Alcohol dehydrogenase is a catalyst of the reduction reaction of polyaromatic aldehyde by reduced form of NADH. Two substrates are specific for isoenzymes class I and II of alcohol dehydrogenase: 4-methoxy-1-naphthaldehyde (for class I ADH isoenzyme) and 6-methoxy-2-naphthaldehyde (for class II ADH isoenzyme). The reaction products are polyaromatic alcohols that exhibit strong fluorescence. The measurements were performed on a Shimadzu RF–5301 spectrofluorophotometer (Shimadzu Europa GmbH, Duisburg, Germany) at excitation wavelenght 316 nm for both substrates and emission of 370 nm for class I and 360 nm for class II isoenzymes (Orywal et al. 2017).

#### Determination of Class III ADH Isoenzyme Activity

The class III ADH isoenzyme is a catalyst of the oxidation reaction of caprylic alcohol. Caprylic alcohol is a substrate for other ADHs as well. The reaction involves nicotinamide adenine dinucleotide which is reduced to NADH. The rate of NADH formation is a measure of isoenzyme activity (Orywal et al. 2017).

#### Determination of Class IV ADH Isoenzyme Activity

The class IV ADH isoenzyme is a catalyst of the reduction reaction of m-nitro-benzaldehyde. The reaction involves a reduced form of nicotinamide adenine dinucleotide that is oxidized to NAD^+^. The NADH decay rate is a measure of isoenzyme activity (Orywal et al. 2017).

#### Determination of Total ALDH Activity

Aldehyde dehydrogenase is a catalyst of the oxidation reaction of polyaromatic aldehyde (6-methoxy-2-naphtaldehyde) by NAD^+^. The resulting product is 6-methoxy-2-naphthoic acid that exhibits strong fluorescence. Fluorescence intensity is a measure of enzyme activity. The fluorescence was read at excitation wavelength 310 and emission wavelength 360 nm (Orywal et al. 2017).

#### Ethics

The research protocol was approved by the Bioethics Committee of the Medical University in Bialystok, Poland (Approval No. R-I-002/181/2014). All patients gave their informed consent to participate in the study.

#### Statistical Analysis

The results were processed statistically in Statistica PL software ver. 10. The *χ*^2^ test revealed that the results did not have normal distribution, and the significance of differences between the compared groups was determined with the Wilcoxon signed-rank test at *p* < 0.05.

## Results

### Serum Activity of Total ADH, ADH Isoenzymes and ALDH in Patients with PBC and in the Control Group

The activity of total ADH, ADH isoenzymes and ALDH in the blood serum of PBC patients is presented in Table 2. The activity of class I and II ADH isoenzymes was higher in the experimental group than in the control group, but a significant difference was observed only in the activity of class I ADH (*p* < 0.001). Class I ADH activity was 4.17 mIU/l in the experimental group and 2.05 mIU/l in the control group. The activity of class III and IV ADH isoenzymes did not differ significantly between experimental group and the control group. Total ADH activity was also significantly higher in PBC patients (1120 mIU/l) than in healthy individuals (520 mIU/l; *p* < 0.001). Serum ALDH activity did not differ significantly between PBC patients and control group subjects.

**Table 2:** Activity of ADH, ADH isoenzymes and ALDH in serum patients with PBC vs control group

	Activity of ADH and ALDH	
	Tested group	Control group
Total ADH (mIU/l)		
Median	1120	520
Range	220–2510	170–1820 (*p* < 0.001)
Total ALDH (mIU/l)		
Median	2.60	2.51
Range	1.10–5.03	0.98–5.03 (*p* = 0.468)
ADH I (mIU//l)		
Median	4.17	2.05
Range	1.25–7.10	0.79–5.24 (*p* < 0.001)
ADH II (mIU//l)		
Median	16.11	13.86
Range	6.07–22.89	5.11–23.72 (*p* = 0.128)
ADH III (mIU//l)		
Median	10.07	10.71
Range	6.32–19.02	6.53–19.23 (*p* = 0.325)
ADH IV (mIU//l)		
Median	4.88	5.03
Range	2.02–10.10	2.12–11.03 (*p* = 0.431)

Substrates for total ADH—p-nitroso-N-N-dimethylaniline; ADH I—4-methoxy-1-naphthaldehyde; ADH II—6-methoxy-2-naphthaldehyde; total ALDH—6-methoxy-2-naphthaldehyde; ADH III—caprylic alcohol; ADH IV—m-nitro-benzaldehyde

### Receiver Operating Characteristic Analysis

The diagnostic value of the parameters showing significant differences was determined at the 97.5 cutoff percentile of the control group. The diagnostic value of class I ADH was set at 5.25 mIU/l, and of total ADH—at 2.52 IU/l. Diagnostic sensitivity and specificity, positive (PPV) and negative (NPV) predictive values are presented in Table 3.

**Table 3:** ROC analysis of ADH I and ADH total of patients with PBC and the controls

	Sensitivity (%)	Specificity (%)	PPV (%)	NPV (%)	AUC (%)
ADH total	63.28	69.01	72.21	68.14	0.714
ADHI	71.42	74.66	79.16	75.54	0.751

The diagnostic sensitivity and specificity of class I ADH and total ADH activity in PBC was compared with the use of receiver operating characteristic (ROC) curves. The area under the ROC curve (AUC) was measured to reveal that class I ADH was characterized by the highest diagnostic accuracy (AUC = 0.751). The AUC for total ADH was somewhat smaller at 0.714.

## Discussion

The diagnosis and treatment of PBC poses a challenge for physicians. The etiology of the disease has not been clearly established, and the existing treatments can only manage the symptoms and slow the progression of PBC. New diagnostic methods are needed to expand our understanding of the mechanisms underlying this disease. Primary biliary cholangitis is considered an autoimmune disease, and it is diagnosed based on elevated AMA levels in the blood serum of patients. Antimitochondrial antibodies are regarded as a serological marker of PBC, and they are detected in less than 1% of the control population. However, the presence of other antibodies should also be considered, including the rheumatoid factor (66%), anti-smooth muscle antibodies (66%), antithyroid antibodies (40%) and antinuclear autoantibodies (30–35%). Anti-Sp100 and anti-gp210 antibodies show the highest specificity (> 95%) for PBC and can be used as diagnostic markers when AMA titers are low. However, these antibodies are characterized by low sensitivity and are identified in only around 25% of PBC patients (Lindor et al. 2009). In approximately 5–10% of the patients, AMA antibodies are absent. The diagnosis of AMA-negative PBC requires an invasive liver biopsy, which shows the typical features of bile duct damage seen in PBC.

The aim of this study was to assess the serum activity of total ADH, ADH isoenzymes and ALDH in patients with PBC. It was found that the class I ADH isoenzyme and/or total ADH can be useful in the diagnosis of PBC because their activity levels differ significantly between PBC patients and healthy individuals. The increase of total ADH activity is a result of the release into the blood of a class I isoenzyme ADH. Class I ADH is the classical liver alcohol dehydrogenase. This class is found up to 95% of total activity in the liver (Chrostek and Szmitkowski 1999). Our study also demonstrates that the activity of class III and IV ADH are not significantly different in the serum of patients with PBC. High activity of class III ADH was characterized in pancreas, and the activity of class IV ADH in the gastric mucosa.

Similar observations were made in a study of AIH, where the activity of class I ADH and total ADH was significantly higher in the serum of AIH patients (Jelski et al. 2018a). It should be noted that both PBC and AIH are autoimmune diseases, and the AIH-PBC overlap syndrome is observed in many patients (Chen et al. 2013). As previously mentioned, ALT and AST activity is fairly normal in classic PBC. However, when serum ALT levels are high (more than fivefold higher than the reference value), the presence of viral and autoimmune comorbidities should be considered (Lindor et al. 2009).

Jelski et al. (2018b) examined the serum activity of total ADH, ADH isoenzymes and ALDH in hepatitis B and C patients (Chrostek and Szmitkowski 1999). The activity of the class I ADH isoenzyme was considerably higher in hepatitis B patients than in the control group, and the activity of class II ADH was elevated in the experimental group, but not significantly higher than in the control group (Chrostek and Szmitkowski 1999). In hepatitis C patients, the activity of class I ADH was significantly higher, and the activity of class II ADH was considerably elevated. In patients with hepatitis B, hepatitis C and autoimmune disorders, an increase in the activity of class I ADH could be associated with considerable hepatocyte damage that accompanies these diseases (Jelski et al. 2018b).

The activity of class I ADH and total ADH also increases in patients with liver metastases. Jelski et al. (2008) found that class I ADH activity was considerably higher in patients with liver metastases than in subjects with primary liver cancer. In our previous study, the serum activity of total ADH, class I ADH and class II ADH was higher in patients with both alcoholic and non-alcoholic fatty liver diseases than in the control group (Jelski et al. 2018a; Wolszczak-Biedrzycka et al. 2022).

It should be noted that class I ADH is localized mainly in liver epithelial cells (95%); therefore, cell damage increases class I ADH activity as well as total ADH activity (Vaglenova et al. 2003). Total ADH activity and class I ADH activity can be used as reliable markers of many liver diseases due to their significant increase in the patients’ blood serum.

Despite the progress in the development of non-invasive diagnostic methods, biopsy is still regarded as the gold standard for diagnosing most liver diseases. As previously mentioned, biopsy is not required for PBC diagnosis, but it may be useful for describing the stage of the diseases. It should be noted that AMA titers are not significantly correlated with any histopathological grading system for assessing liver biopsies in PBC patients. Liver biopsy can be used to further substantiate the diagnosis, assess disease severity, and differentiate PBC from other liver diseases (e.g., cirrhosis). An ultrasonic examination of the abdominal cavity is useful for evaluating overall liver health, but it does not support the identification of changes characteristic of PBC (Gatselis et al. 2013). However, an ultrasound scan can rule out mechanical obstruction of bile flow and confirm the presence of focal changes (Lindor et al. 2009). Efforts are being made to develop new methods for early PBC diagnosis. Previous studies demonstrated that immunostaining for biliary keratin 19 (K19) may reveal early changes that are associated with PBC, namely the loss of the canals of Hering. These structures connect bile ductules lined by cholangiocytes and hepatocytes with interlobular bile ducts (Chen et al. 2013). The results of our study indicate that the serum activity of total ADH and class I ADH can be used as non-invasive markers of liver damage in PBC as well as non-invasive determination of AMA. In addition, the combined determination of ADH I and AMA antibodies should be considered.

## Conclusions

The study demonstrated that a considerable increase in the activity of class I ADH and total ADH in the blood serum of PBC patients results from the release of the class I ADH isoenzyme from hepatocytes during disease development. The present findings suggest that the total ADH activity or class I ADH activity can be used as markers for diagnosing the progression of PBC.

## Data Availability

Data are availability in corresponding author.
